# Healthy Neighborhoods: Walkability and Air Pollution

**DOI:** 10.1289/ehp.0900595

**Published:** 2009-07-20

**Authors:** Julian D. Marshall, Michael Brauer, Lawrence D. Frank

**Affiliations:** 1 Department of Civil Engineering, University of Minnesota, Minneapolis, Minnesota, USA; 2 School of Environmental Health and; 3 School of Community and Regional Planning, University of British Columbia, Vancouver, British Columbia, Canada

**Keywords:** air quality, built environment, exercise, infill, pedestrian friendliness, physical activity, sprawl, traffic, urban design, urban environmental health, vehicle emissions

## Abstract

**Background:**

The built environment may influence health in part through the promotion of physical activity and exposure to pollution. To date, no studies have explored interactions between neighborhood walkability and air pollution exposure.

**Methods:**

We estimated concentrations of nitric oxide (NO), a marker for direct vehicle emissions), and ozone (O_3_) and a neighborhood walkability score, for 49,702 (89% of total) postal codes in Vancouver, British Columbia, Canada. NO concentrations were estimated from a land-use regression model, O_3_ was estimated from ambient monitoring data; walkability was calculated based on geographic attributes such as land-use mix, street connectivity, and residential density.

**Results:**

All three attributes exhibit an urban–rural gradient, with high walkability and NO concentrations, and low O_3_ concentrations, near the city center. Lower-income areas tend to have higher NO concentrations and walkability and lower O_3_ concentrations. Higher-income areas tend to have lower pollution (NO and O_3_). “Sweet-spot” neighborhoods (low pollution, high walkability) are generally located near but not at the city center and are almost exclusively higher income.

**Policy implications:**

Increased concentration of activities in urban settings yields both health costs and benefits. Our research identifies neighborhoods that do especially well (and especially poorly) for walkability and air pollution exposure. Work is needed to ensure that the poor do not bear an undue burden of urban air pollution and that neighborhoods designed for walking, bicycling, or mass transit do not adversely affect resident’s exposure to air pollution. Analyses presented here could be replicated in other cities and tracked over time to better understand interactions among neighborhood walkability, air pollution exposure, and income level.

## Air Pollution, Physical Activity, and Neighborhood Design

The built environment affects public health in many ways (Frumkin et al. 2004), depending on the interplay between factors such as community design, travel patterns, physical activity, transportation safety, and air and water pollution. This study investigated interactions between *a*) walkability, a measure of how conducive the built environment is to walking and that predicts physical activity and active transportation (Frank et al. 2005; Owen et al. 2004; Sallis et al. 2004), and *b*) exposure to outdoor air pollution, which is associated with a wide array of negative health impacts.

Physical inactivity and outdoor urban air pollution are two of the top 15 global causes of health impairment (Ezzati et al. 2002; Hill et al. 2003). Ozone (O_3_) (Bell et al. 2006; Jerrett et al. 2009), vehicle exhaust (Brauer et al. 2008; Kim et al. 2004), and within-city contrasts in other air pollutants for which traffic is a major contributor (Brunekreef and Holgate 2002; Mokdad et al. 2004; Vedal et al. 2003) are associated with many adverse health outcomes, including cardiopulmonary mortality (Beelen et al. 2008; Nafstad et al. 2004; Pope et al. 2002), atherosclerosis (Hoffmann et al. 2007; Künzli et al. 2005), impaired lung development in children (Gauderman et al. 2007), asthma and asthma exacerbations (Brauer et al. 2007; Gauderman et al. 2005; Trasande and Thurston 2005), reduced lung function (Brunekreef et al. 1997; Kulkarni et al. 2006), cardiac arrhythmia (Peters et al. 2000), and preterm and low-birth-weight babies (Brauer et al. 2008; Parker et al. 2005; Šrám et al. 2005; Wilhelm and Ritz 2003). Inactivity and insufficient activity (< 2.5 hr/week of moderate-intensity activity, or < 4,000 kJ/week) have been causally linked with heart disease, several cancers, diabetes, and other adverse health impacts and are associated with high body mass index (BMI; overweight and obesity), which can lead to additional effects on health (Ezzati et al. 2002). In polluted U.S. cities, the mortality risk from particulate air pollution is comparable to that for obesity (grade 1 or 2), but less than that for extreme obesity (grade 3) (Pope et al. 2002). Reducing the average energy imbalance (caloric intake minus metabolic activity) among persons in the United States by approximately 100–165 kcal/day would prevent average weight gain (~ 1 kg/year) (Hill et al. 2003; Wang et al. 2006), which suggests that moderate daily exercise—as little as two or three 10-min walking trips, such as to a bus stop or grocery store—could provide major public health benefits.

“Walkability” of a neighborhood measures whether community design encourages or inhibits walking (Frank and Engelke 2001; Frank et al. 2004; Gordon-Larsen et al. 2006; Handy et al. 2002; Li et al. 2005). For example, lack of a sidewalk can make walking unsafe, and a disconnected street network can discourage walking. Conversely, having retail stores close to where people live and providing connected streets increases the likelihood that a person will incorporate walking into daily routines (Frank et al. 2005; Moudon et al. 2007).

Neighborhood design—for example, the layout of buildings, land uses, and streets—can influence walking and other exercise activities, BMI, and overall health ratings, as well as air pollution emissions and exposures [Cervero and Duncan 2003; Ewing et al. 2003; Frank et al. 2004, 2005; Kelly-Schwartz et al. 2004; Owen et al. 2004; Smith et al. 2008; U.S. Environmental Protection Agency (EPA) 1999] (Table 1). The American Academy of Pediatrics (Committee on Environmental Health 2004), the U.S. Centers for Disease Control (Kochtitzky et al. 2006; Martin and Carlson 2005), the World Health Organization (Edwards and Tsouros 2006; WHO 2006), and others (Dearry 2004; Jackson and Kochtitzky 2001) have called for research on how city design affects walking and other exercise and people’s exposure to air pollution. An important goal is using neighborhood design as a tool for creating cleaner, healthier urban environments.

Our analyses identify neighborhoods that do especially well (or poorly) for both issues (walkability, air pollution). To our knowledge, this study is the first to compare quantitative estimates for these two neighborhood-scale environmental health attributes. Our findings demonstrate important health impacts of spatial exposure to the built environment.

## Materials and Methods

We investigated air pollution concentrations and walkability in Metro Vancouver, which is a coastal urban region of 2.2 million people in southwest British Columbia, Canada (average density, 760 people/km^2^). Pollution and walkability were estimated using geographic information system (GIS) mapping software (ArcGIS; ESRI, Redlands, CA, USA). To our knowledge, our study is the first to quantitatively assess the spatial intersection of walkability and air pollution (Frank and Engelke 2005).

Vancouver, which sits prominently at the top of international rankings of livable cities (The Economist 2009), is a useful study region because of the wide range in walkability levels. It is often cited as a well-planned city with walkable neighborhoods and comparatively clean air. However, the suburbs in Vancouver are as unwalkable as the suburbs found in typical sprawling regions (Montgomery 2006).

Concentrations of nitric oxide (NO), which is an indicator of traffic exhaust and of O_3_, a regional, secondary pollutant, were estimated for 49,702 (89%) of the 56,099 postal codes in Metro Vancouver (Marshall et al. 2008). Walkability estimates were then generated for those postal codes. Postal codes in this region are typically one city block-face (one side of a block) or smaller (Metro Vancouver’s average postal code size is 39 people, or 0.05 km^2^). Because postal codes have roughly equal populations, analyses by postal code are roughly population-weighted analyses. The Canadian census (Statistics Canada 2004) provides a 1-to-5 low-to-high measure of affluence [quintile of annual income per person equivalent (QAIPPE)] for each postal code.

### Walkability

Walkability captures the proximity between functionally complementary land uses (live, work, play) and the degree of route directness or connectivity between destinations (Forsyth and Southworth 2008; Moudon et al. 2006). Our walkability estimates incorporated four parameters (Frank et al. 2009; Leslie et al. 2007): *a*) net residential density (*D*), the number of dwelling units per square kilometer of residential land; *b*) intersection density (*I*), the number of intersections per square kilometer; *c*) retail floor area ratio (*R*), the retail shop floor-area divided by retail land area; and *d*) land-use mix (*M*), the evenness (i.e., equality) of floor space among categories of land use. We calculated each parameter for a 1-km network buffer around each postal code (Frank et al. 2009) and then a relative walkability score (*W*; unitless) for that postal code as


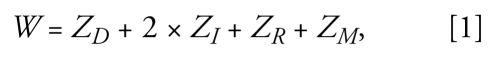


where *Z_D_*, *Z_I_*, *Z_R_*, and *Z_M_* are statistical *Z*-scores [unitless (mean ≈ 0; SD ≈ 1)] for *D*, *I*, *R*, and *M*, respectively. Values for *I* are large where streets are well connected (e.g., a grid) and small where streets are poorly connected (e.g., cul-de-sacs). Values for *R* are large for multistoried retail buildings with little surface parking and small for one-story retail buildings with large parking lots (“big-box retail”). Values for *M* are large where land uses such as residential, retail, office, and entertainment are highly mixed, and small where they are spatially homogeneous. Values for *M* are calculated based on building area within 27 land-use categories. The data used in Equation 1 were derived from three sources: *a*) locations for postal code centroids (Postal Code Conversion File, version 4D) and demographic information such as QAIPPE are from the 2001 Canadian Census (Statistics Canada 2004), *b*) street network data are from the 2001 CanMap (DMTI Spatial, Markham, Ontario, Canada), and *c*) land-use data are from the 2001 British Columbia Property Assessment (BC Assessment, Victoria, British Columbia, Canada) (Setton et al. 2005).

Parameters in Equation 1 were selected because they have been found in many studies to be predictors of travel patterns and of walking in particular (Engelke et al. 2003; Ewing et al. 2003; Frank et al. 2005, 2006; Lee and Moudon 2006). For example, in Seattle, a 5% increase in the Equation 1 walkability score is associated with a 33% increase in the proportion of people who reported that they walked during a 2-day period (Frank et al. 2006).

### Air pollution

For each postal code, we evaluated the annual average concentrations of two pollutants: NO concentrations were estimated using land-use regression (LUR) (Henderson et al. 2007), and O_3_ concentrations were estimated using spatial interpolation of summer-only (May–September) monitoring data (Vedal et al. 2003). We employed inverse-distance weighted average of the three nearest monitors (Marshall et al. 2008); among the postal codes, the mean distance to the nearest O_3_ monitor is 3.7 km. These two approaches—LUR for NO and interpolation for O_3_—were selected because extant estimates are available for Metro Vancouver. In addition, the approaches match well with each pollutant’s spatial variability: generally, NO concentrations vary over short spatial scales (roughly one or a few city blocks), whereas O_3_ varies over long spatial scales (suburbs vs. urban core) (Marshall et al. 2008).

NO was chosen because it is a primary vehicle-related pollutant and therefore serves as a marker for freshly emitted traffic exhaust, including both gasoline and diesel vehicles. O_3_ offers a useful comparison with NO because it is a secondary pollutant (i.e., formed in the atmosphere rather than emitted directly), so high concentrations tend to occur regionally downwind of the highest density areas (e.g., in suburbs). High O_3_ concentrations occur in summer.

We have previously described and validated the LUR model (Henderson et al. 2007) and compared it against interpolation and mechanistic air dispersion models (Marshall et al. 2008). LUR is a hybrid empirical-statistical approach that combines concentration measurements with GIS maps, thereby offering a high degree of spatial resolution. Briefly, 116 passive NO samplers were deployed for two 14-day periods at 116 sites in the study area. Mean concentrations during these two periods were successfully validated against annual averages from regulatory monitoring network data (Henderson et al. 2007; Marshall et al. 2008). For each measurement site, 55 variables were generated in GIS. Linear regression models of NO were built with the most predictive covariates. The model has an *R*^2^ of 0.62 and includes as covariates the number of major roads within 100- and 1,000-m radii circular buffers of the measurement sites, the number of secondary roads within a 100-m buffer, the population density within a 2,500-m radius, and elevation. As described previously (Marshall et al. 2008), model–measurement comparisons for NO at monitoring station locations indicate reasonable to good agreement (mean bias, absolute bias, and error, 29%, 42%, and 1.6 μg/m^3^, respectively; model–measurement correlation, 0.7). Bias and absolute bias levels meet the goals (30% and 50%, respectively) and criteria (60% and 75%, respectively) suggested by Boylan and Russell (2006) for particulate matter concentration, but they do not meet the regulatory guidance (15% and 35%, respectively) for peak O_3_ concentrations (U.S. EPA 1991).

## Results

**Figure 1:**
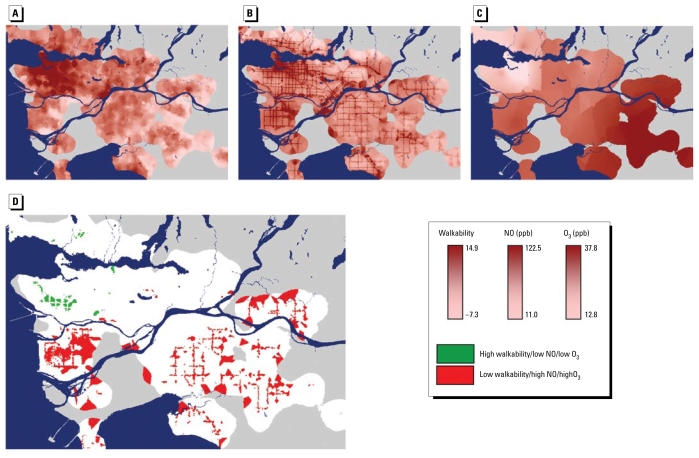
Maps of walkability (*A*) and pollutant concentrations of (*B*) NO and (*C*) O_3_, (*D*) “Sweet-spot” and “sour-spot” postal codes based on tertiles.

In Figure 1 and Table 2, we present walkability levels and air pollution concentrations. Mean values are 0.33 for walkability (unitless), 32.1 for NO (micrograms per cubic meter), and 27.7 for O_3_ (micrograms per cubic meter). Table 2 also presents results from two spatial analyses: distance from city center (the Vancouver courthouse) and spatial length scale for variability. The former analysis reports the median distance between each postal code and downtown Vancouver, stratifying postal codes by tertile of walkability, NO, or O_3_. The latter analysis measures the change in location needed to observe a modest change, in this case, one-half of the overall spatial SD in each parameter (Marshall 2008).

Suburbs and exurbs tend to have high concentrations of O_3_, yet low levels of NO and walkability. The reverse holds for downtown areas. Those findings are expected: walkability parameters in Equation 1 are higher downtown than in the suburbs [see Supplemental Material, Figure 1, available online (doi:10.1289/ehp.0900595.S1 via http://dx.doi.org/)], and NO concentrations are elevated near the high density of vehicle emissions downtown. In contrast, O_3_ is a secondary pollutant formed from chemical reactions of nitrogen oxides (NO_x_) and volatile organic compounds (VOCs) in the presence of sunlight; as O_3_ forms, air migrates. In addition, NO reacts with and removes (“titrates”) O_3_, thereby reducing O_3_ concentrations where traffic emissions (and NO concentrations) are high.

NO has a relatively short length scale (0.4 km), which indicates high spatial heterogeneity: changing location by a small distance can yield a comparatively large change in NO concentration. O_3_ has a longer length scale (8 km) because it is a regional pollutant and because estimations are derived from spatial interpolation of the monitoring data. The length scale for walkability is between that for NO and O_3_. Length scales (kilometers) for the Equation 1*Z*-scores vary significantly (and are between the NO and the O_3_ length scales): *Z_D_*, 5.6; *Z_I_*, 0.8; *Z_R_*, 4.8; *Z_M_*, 0.6. Variability among length scales highlights the possibility that certain areas might avoid high pollution levels yet have moderate or high walkability.

**Figure 2:**
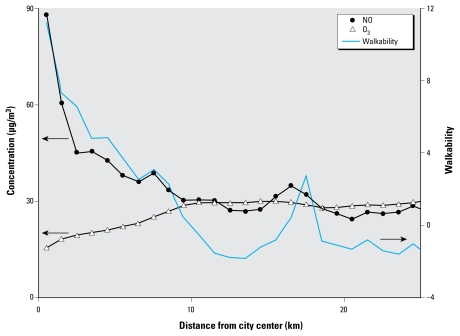
Mean value for three parameters as a function of distance from city center (Vancouver courthouse). To avoid oceans and mountains, only postal codes in the quadrant southeast of the city center are included.

Figure 2 presents average values for parameters as a function of distance from downtown. Walkability and NO levels decline sharply in the first 6 km from downtown, whereas O_3_ increases consistently along the urban-to-rural gradient. The high walkability area 16–19 km from downtown represents older satellite cities (e.g., New Westminster). The slight increase in walkability approximately 4 km from downtown derives from increases in intersection density. Importantly, locations approximately 4–8 km from downtown avoid the peak concentrations of NO and O_3_, yet they still have above-average walkability levels.

A plot analogous to Figure 2, but for the Equation 1*Z*-scores [see Supplemental Material, Figure 1(doi:10.1289/ehp.0900595. S1)], reveals that intersection density has an overall maximum approximately 5 km from downtown and a local maximum approximately 18 km from downtown (which causes the walkability increase at 16–19 km in Figure 2). In contrast, for the remaining three *Z*-scores (net residential density, retail floor area ratio, land-use mix), values are high downtown, decline rapidly for the first 5–10 km, and then are relatively constant (roughly between 0 and −0.5) for distances > 10 km from downtown.

Among the Equation 1*Z*-scores, NO concentrations are most correlated with residential density (Pearson *r* = 0.53) and least correlated with intersection density (*r* = 0.24). This finding suggests that “cars follow people, not roads”: neighborhoods with moderate or low residential density may avoid traffic exhaust yet still offer high intersection density (thereby improving walkability). Consistent with Figure 1, walkability is correlated with NO concentrations (*r* = 0.49) and inversely correlated with O_3_ concentrations (*r* = −0.70). The Supplemental Material contains additional pairwise comparisons and statistical models of the three attributes (walkability, NO, and O_3_), as well as correlations between those attributes and traffic counts [modeled peak morning traffic—automobile and truck—within several radii, 100–1,000 m, of each postal code centroid (Henderson et al. 2007)]. As expected, traffic is correlated with NO (*r* = 0.37–0.68) and walkability (*r* = 0.18–0.64) and inversely correlated with O_3_ (*r* = −0.14 to −0.59); correlations are larger in magnitude for automobile traffic than for truck traffic and for larger radii than for smaller radii. Among Equation 1*Z*-scores, traffic is least correlated with intersection density and most correlated with residential density (automobile traffic) and land-use mixing (truck traffic).

**Figure 3:**
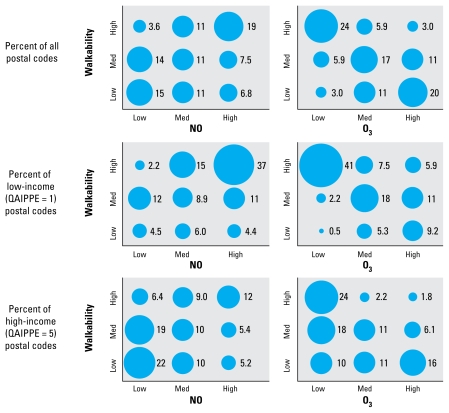
Percentage of postal codes in each walkability and pollution tertile. For example, low walkability and low ozone is found in 0.5% of low-income postal codes and in 10% of high-income postal codes. Values in each panel total 100%.

Figure 3 presents distributions of postal codes by walkability and pollution tertile. Fewer than 4% of postal codes have high walkability yet low NO concentrations, whereas 24% of postal codes have high walkability and low O_3_ concentrations. (If attributes were uncorrelated, each entry in Figure 3 would be 11%.) The proportion of postal codes with low walkability and low NO is 6 times greater for highest-income than for lowest-income postal codes (22% vs. 4%). This finding is consistent with expectations, because on average neighborhoods are more affluent in suburbs than near the city center. Affluent postal codes tend to be less walkable and have lower NO; the reverse holds for poor postal codes.

Figure 3 also illustrates postal codes in the most and least desirable tertile for each attribute: “Sweet-spot” postal codes are high walkability, low pollution; “sour-spot” postal codes are the opposite (low walkability, high pollution). We visited several sweet-spot neighborhoods. Our informal examination indicated urban-form attributes consistent with results presented above: high street connectivity, mixed land uses nearby, absence of large parking lots near retail space, sidewalks, and, in general, active streets with many walkers.

Table 3 presents distributions of postal codes by income for specific walkability and pollution tertiles. Although NO and walkability values follow relatively monotonic patterns with income, O_3_ does not. High O_3_ occurs most commonly for middle- and upper-middle income postal codes, and low O_3_ occurs most commonly for lowest and highest income postal codes.

The prevalences of overall sweet- and sour-spot postal codes in Table 3 are 1.7% and 4.6%, respectively. [If the attributes were uncorrelated, the prevalence would be 3.7% (i.e., 3^−3^).] Sweet-spot postal codes are heavily skewed toward high incomes (68% are highest income, QAIPPE = 5; only 3% are lowest income, QAIPPE = 1) and tend to be near but not at the city center (mean ± SD distance from city center, 6 ± 1 km). Sour-spot postal codes are predominantly from the middle three income quintiles and tend to be spread far from the city center (mean ± SD distance from city center, *r*= 22 ± 11 km).

For postal codes with high walkability, we compared Equation 1*Z*-scores for low pollution versus not low pollution and found that the former group has a higher average score for *Z_R_* (1.7 vs. 1.0) and lower scores for the three other parameters (*Z_I_*, 0.7 vs. 1.1; *Z_M_*, 0.5 vs. 0.9; *Z_D_*, −0.3 vs. 0.3). Thus, what separates sweet-spot postal codes from other highly walkable postal codes is proximity to shops with limited parking per retail floor area.

As discussed below, we investigated differences in air pollution and walkability for neighborhoods near versus not near mass transit by comparing attributes for postal codes inside and outside of 0.25-, 0.5-, and 1-mile circular buffers around light-rail (SkyTrain) stations. On average, the near-station postal codes tend to have higher walkability [mean difference (unitless) ± SD, 3 ± 1], higher NO (30–40%), and lower O_3_ (10%) than do areas not near rail stations. The difference in walkability is mainly attributable to differences in net residential density and land-use mixing.

## Discussion

We explored urban spatial patterns for three environmental health attributes (walkability; NO, a primary pollutant with high concentrations near traffic and other combustion sources; and, O_3_, a secondary pollutant). Our results help elucidate spatial variations in community design, exposure to air pollution, and income. The methods presented provide new planning tools that can help identify where increased levels of physical activity may occur because of higher levels of walkability and where traffic emissions and O_3_ are least and most heavily concentrated. Walkability and air pollution have independently been associated with several adverse health outcomes, including physical inactivity, heart disease, mortality, and atherosclerosis, but health studies have not yet investigated interactive or joint effects. Our investigation builds on previous research and highlights the complexities of built environment and health relationships.

We observed spatial differences among the three attributes. On average, conditions are better in the urban core than in the suburbs for O_3_ and walkability; the opposite is true for NO. We further identified neighborhoods that had reasonable levels for walkability and pollution. Sweet-spot locations, representing less than 2% of postal codes, are concentrated near but not at the city center. Most of them are higher income, indicating that they are highly desirable locations. In contrast, sour-spot postal codes are far from the city center, are spread more widely, and are occupied mostly by middle-income groups, although the degree of income segregation is less than for the sweet spots. Our finding that income is correlated with O_3_ concentrations but is inversely correlated with primary pollutant (NO) concentrations is consistent with similar findings in Southern California (Marshall 2008; Marshall et al. 2006; Morello-Frosch et al. 2001, 2002). Our results add to the literature on environmental justice aspects of air pollution (Brulle and Pellow 2006; O’Neill et al. 2003), including on exposure to traffic-related pollution (Buzzelli and Jerrett 2007; Havard et al. 2009; Houston et al. 2008; Kingham et al. 2007). Results in North America suggest that for NO and other primary pollutants, low-income and nonwhite populations face a disproportionate share of the burden of urban air pollution.

Length-scale calculations highlighted that the aspects of the built environment studied here exhibit differing spatial patterns. For example, intersection density is a somewhat local attribute (shorter length scale), whereas retail floor area ratio is more regional. These age findings are important for several reasons. They suggest that when considering multiple attributes of the built environment, analysts may be able to identify areas trading off one attribute for the other, as well as sweet- and sour-spot locations. This attribute is useful for policy evaluation and for identifying locations meriting further investigation. In addition, people travel a finite distance each day. [In one study, which was not conducted in Vancouver, researchers found that the typical daily maximum distance from home is approximately 5 km (Marshall et al. 2006).] During each travel day, individuals may experience a wider range of values for some aspects of the built environment than for others. For example, based on the length-scale results presented above, an individual willing to walk 2 km is likely to reach locations with different levels of land-use mixing but with similar retail floor area ratios.

Policies designed to improve one attribute may hinder other attributes. For instance, modest reductions in NO emissions can increase O_3_ locally (because NO titrates O_3_) and either increase or decrease O_3_ regionally (depending on whether O_3_ chemistry is limited by NO_x_ or VOCs). In areas with low walkability, people often have high levels of driving and of vehicle emissions per person (Frank et al. 2000, 2006; Frumkin et al. 2004), but if activities and emissions are dispersed, then concentrations of vehicle emissions may be low (Marshall et al. 2005). Conversely, walkable neighborhoods may exhibit reduced per-capita vehicle use and emissions (Frank and Engelke 2005; Frank et al. 2000, 2006) yet elevated traffic congestion, emissions, and concentrations if activities are highly concentrated. Ideally, one would understand all important impacts before recommending a policy action such as alternative growth patterns or transportation investments. Our research highlights that high NO exposures may occur where physical activity is encouraged through active transportation and in low-income areas. More work is needed to understand how to avoid that outcome, especially for susceptible subpopulations such as youth and the elderly. Policy options include siting residential buildings (especially schools, daycare centers, and assisted living facilities) back from major transportation corridors, rules or incentives to reduce high-emitting vehicles in urban centers, indoor air cleaners, and high-rise buildings that distance inhabitants from ground-level pollution. Shifts in the built environment can provide an intervention strategy for improving environmental health. Policies that provide affordable housing in walkable areas with cleaner air can help to offset the trend of low-income households experiencing worse than average primary air pollution; access to employment and other opportunities is an important aspect of overcoming disadvantages faced by traditionally underserved groups. Our approach would allow policy makers to evaluate and spatially optimize changes to the built environment that would yield the greatest health benefit per dollar.

Walkable neighborhoods (and also neighborhoods served by mass transit) may allow people to reduce their daily travel distance, thereby decreasing vehicle emissions of NO and other O_3_ precursors. Improving air pollution and walkability will require changes in technologies, such as reducing emissions from motor vehicles, and also in urban design (e.g., land use mixing, mass transit).

Our results presented above indicate that near-transit neighborhoods are more walkable, have lower O_3_ concentrations, and have somewhat higher (30–40%) NO concentrations than do other neighborhoods. The influence of building configuration on air pollution, although not directly explored here, may be an important aspect of this comparison. The increased density near rail stations is often accommodated via taller buildings. High-rise buildings elevate occupants above ground-level emissions. For primary pollutants, concentrations aloft (> 10–25 m, or three to five stories; Zhou and Levy 2008) can be several times lower than at ground level (Väkevä et al. 1999; Zoumakis 1995), which could more than compensate for the 30–40% ground-level NO concentration difference identified here. However, the urban street canyons formed by tall buildings reduce dilution rates and increase near-ground NO concentrations (Chan et al. 2002, 2003; Zhou and Levy 2008). In contrast, because of O_3_ titration by ground-level NO emissions in downtown areas, O_3_ concentrations are often greater aloft than at ground level (Baik et al. 2007; Costabile and Allegrini 2007; Garmory et al. 2009; Wöhrnschimmel et al. 2006).

Additional limitations of the present study include the following: the walkability measure lacks data on sidewalks and parks; O_3_ concentration estimates do not include on-roadway O_3_ titration by NO; and, although the walkability score has been correlated with actual walking levels in several other cities, we have not yet completed that research in Vancouver (excluding our informal field examinations). Although this study focuses on neighborhood design (i.e., urban form; see Equation 1) (Rodriguez and Joo 2004) as a predictor of walking levels (e.g., trip frequency, duration, or distance; percentage of people walking more than a threshold), other factors are also important, including, weather, topography, crime, pedestrian safety, noise, pollution, and personal preferences (Bagley and Mokhtarian 2002; Frank et al. 2007). Similarly, several factors affect the relationship between urban layout and pollutant emissions and concentrations. Limitations in extrapolating from our results to other situations include that other pollutants may not track spatially with NO and O_3_. Unique characteristics of Vancouver include the following: Vancouver is in a coastal air shed bounded by mountains, winds are predominantly from ocean to land, and walkability is relatively high when compared with other North American cities. In this study, we evaluated spatial variability, but future research is needed that considers temporal variability such as diurnal, weekly, and seasonal variations in NO and O_3_ concentrations and walkability and changes over time in urban form. Future research could address these limitations, investigate ways to target or optimize built-environment interventions, and document changes over time or measure the effectiveness of specific policies or actions. Other research could also include data on actual physical activity levels and pollutant exposures across urban form and address the health trade-offs between exposure to air pollution and physical activity for different age and income groups. Comparative research that documents the disparities in air-pollution exposure and walkability across income groups, while also identifying and testing strategies for improvement, is needed. Finally, an important next step toward maximizing the ability of urban form to impact health is the development and refinement of trip-planning tools that incorporate the various health-related attributes to allow individuals to reduce exposures during travel. Cycle Vancouver (2007) offers a first step toward this type of tool.

## Conclusion

Our investigation explores potential environmental health impacts of neighborhood design. We found that neighborhoods with high walkability tend to have high levels of primary traffic-related pollution (NO) but low concentrations of O_3_. Attributes of the built environment evaluated here have differing spatial patterns, but all exhibit an urban–rural gradient. High-walkability and high-NO neighborhoods tend to be low-income neighborhood, whereas neighborhoods with high O_3_ tend to be middle income. Neighborhoods that exhibit low pollution and high walkability are rare and tend to be high income and located near to but not at the city center. Neighborhoods with high pollution and low walkability are far from the city center.

The environmental attributes we studied are associated with several adverse health outcomes. The results emphasize that various aspects of environmental quality exhibit differing spatial patterns. Our analyses could usefully be applied to other cities, employed to track changes over time resulting from urban development and redevelopment, or used to design areas that are low in pollution exposure and that promote physical activity through increased walkability.

## Figures and Tables

**Table 1:** Sample of recent findings relating urban design, environment, and health in the United States.

Study location(s)	Findings
San Diego, CA; Montgomery County, MD; West Palm Beach, FL	Vehicle kilometers traveled (VKT) is 40–50% lower, and emissions of carbon dioxide and of NO_x_ are ~50% lower for new residences in already built-up areas (“infill development”) than for “green-field” development (suburbs/exurbs) (U.S. EPA 1999)
San Francisco, CA	Factors observed to induce nonmotorized travel include well-connected streets, small city blocks, mixed land uses, and close proximity to retail activities (Cervero and Duncan 2003)
448 counties and 83 metropolitan areas	Sprawl reduces walking levels and may increase BMI (Ewing et al. 2003)
Atlanta, GA	Likelihood of obesity decreases 5% for each additional kilometer walked per day, increases 6% for each additional hour spent in a car per day, decreases 12% for a 1-quartile increase in land-use mixing (Frank et al. 2004)
29 metropolitan areas	Health ratings are high in locations with high accessibility and with gridded street networks but are low in high-density areas. (Health ratings are higher in high-density areas with accessible gridded streets than in low-density areas with nonaccessible nongridded streets) (Kelly-Schwartz et al. 2004)
Atlanta, GA	Land-use mixing, residential density, and intersection density are correlated with minutes of moderate physical activity per day. Based on objectively measured (accelerometer-based) activity, individuals were 2.4 times more likely to meet recommended activity levels (30 min/day) in the highest walkability quartile than in the lowest (Frank et al. 2005)
Salt Lake City, UT	Risk of obesity is lower among persons living in older and in more pedestrian-friendly neighborhoods. Differences in body weight between most- and least-walkable neighborhoods was ~ 8 pounds (Smith et al. 2008)

**Table 2:** Summary of walkability and air pollution results.

Measure	Walkability	NO	O_3_
Mean ± SD (median)^a^	0.33 ± 3.34 (−0.59)	32.1 ± 14.6 (27.9)	27.7 ± 4.78 (28.7)
Interquartile range^a^	−1.96 to 2.35	23.6 to 35.6	23.8 to 30.5
Median distance from city center^b^ (km), by tertile^c^			
Lower	23 (25)	19 (22)	5.2 (7.4)
Middle	17 (21)	17 (19)	17 (20)
Upper	7.0 (6.7)	11 (8.9)	25 (27)
Length scale of spatial variability^d^ (km)	1.5	0.4	7.9

a Units for NO and O_3_ concentrations are μg/m3; walkability is unitless.

b Values are for all postal codes (values in parentheses are for postal codes in the southeast quadrant from city center only; avoids ocean and mountains).

c Refers to postal codes in the given tertile for each parameter.

d For each parameter (walkability, NO, or O_3_), the distance at which the average parameter difference between two postal codes is equal to half of the overall spatial standard deviation for the parameter (Marshall et al. 2008).

**Table 3:** Pollution and walkability (W) tertiles: prevalence, by income quintile and average distance from city center (Vancouver courthouse).

Measure	All postal codes (100%)	Low NO (33%)	Low O_3_ (33%)	Low W (33%)	High NO (33%)	High O_3_ (33%)	High W (33%)	Low NO, high W (4%)	Low O_3_, high W (24%)	Low NO, low O_3_, high W (2%)	High NO, high O_3_, low W (5%)
Prevalence by income quintile (QAIPPE)											
1 (low)	1.0	0.55	1.30	0.45	1.55	0.78	1.62	0.62	1.67	0.18	0.66
2	1.0	0.78	0.82	0.83	1.12	1.04	1.13	0.72	1.01	0.23	1.18
3	1.0	1.03	0.64	1.07	0.90	1.17	0.85	0.98	0.74	0.40	1.20
4	1.0	1.13	0.70	1.42	0.83	1.28	0.66	0.78	0.70	0.69	1.35
5 (high)	1.0	1.42	1.56	1.12	0.69	0.70	0.84	1.81	0.98	3.23	0.58
Average distance from city center											
Mean distance (km)	16	19	5.2	23	11	25	7.0	10	4.5	6.2	22
Coefficient of variability	70%	54%	42%	48%	89%	42%	84%	51%	45%	18%	48%

Values in each column present the relative prevalence of postal codes with that income quintile, normalized to 1.0 = prevalence in Metro Vancouver. For example, from the first column, lowest income postal codes are half (55%) as prevalent among low-NO postal codes as they are in Metro Vancouver, and highest income is 42% more common among low-NO postal codes than overall in Metro Vancouver. The column heading indicates percentage of the 49,702 Metro Vancouver postal codes represented by that column. For example, 33% of postal codes are in the low-NO tertile; 4% of postal codes are both low NO and high walkability.
